# Correction: Laiton-Bonadiez et al. Deep 3D Neural Network for Brain Structures Segmentation Using Self-Attention Modules in MRI Images. *Sensors* 2022, *22*, 2559

**DOI:** 10.3390/s26031030

**Published:** 2026-02-05

**Authors:** Camilo Laiton-Bonadiez, German Sanchez-Torres, John Branch-Bedoya

**Affiliations:** 1 Facultad de Minas, Universidad Nacional de Colombia, Medellín 050041, Colombia; jwbranch@unal.edu.co; 2 Facultad de Ingeniería, Universidad del Magdalena, Santa Marta 470001, Colombia; gsanchez@unimagdalena.edu.co

**Text Correction**

In the original publication [1], there was an error. A correction has been made to Section 1, paragraph 11. The contents “Recently, deep learning has become an area of interest in the scientific community due to the important results that have been achieved in multiple disciplines [39–43]” have been replaced by “Recently, deep learning has become an area of interest in the scientific community due to the important results that have been achieved in multiple disciplines [39–41]”.

**References**

References [42,43] have been removed. With this correction, the order of some references has been adjusted accordingly.

The authors state that the scientific conclusions are unaffected. This correction was approved by the Academic Editor. The original publication has also been updated.
